# Morphological Characteristics, Sediment Grain Size, and Spatial Distribution Patterns of *Caragana tibetica* Nabkhas in Desert Steppe

**DOI:** 10.3390/plants15081235

**Published:** 2026-04-17

**Authors:** Yanlong Han, Min Han, Yong Gao, Minghui He, Zhenliang Wu, Wenyuan Yang

**Affiliations:** 1 College of Desert Control Science and Engineering, Inner Mongolia Agricultural University, Hohhot 010018, China; 18804896323@126.com (Y.H.); 13314729077@163.com (M.H.); 15049399074@163.com (W.Y.); 2 State Key Laboratory of Water Engineering Ecology and Environment in Arid Area, Inner Mongolia Agricultural University, Hohhot 010018, China; 3 Inner Mongolia Hangjin Desert Ecological Position Research Station, Ordos 017400, China; 4 Inner Mongolia Autonomous Region Forestry and Grassland Monitoring and Planning Institute, Hohhot 010020, China; spring0455@163.com; 5 Baotou Wetland Protection Center, Baotou 014000, China; ll13947864131@163.com

**Keywords:** *Caragana tibetica*, nabkha morphological characteristics, grain size characteristics, spatial pattern, desert steppe

## Abstract

Nabkhas are a common type of biogenic aeolian landform in arid and semi-arid regions. Their morphological characteristics, surface sediment grain size composition, and spatial distribution patterns can, to some extent, be associated with the interactions between vegetation and the aeolian environment. In this study, nabkhas formed around *Caragana tibetica* shrubs in the desert steppe of Damao Banner, Inner Mongolia, were selected as the research object. Based on field investigations, UAV image identification, grain size analysis, and spatial point pattern analysis, the characteristics of nabkhas were comparatively analyzed among a control plot without shrubs (CK) and three shrub-covered plots: a low coverage plot (LCP), a medium coverage plot (MCP), and a high coverage plot (HCP). The results showed that (1) some morphological parameters of nabkhas varied among plots with different vegetation cover, but the responses of various indicators were not entirely consistent. The MCP exhibited relatively higher values in indicators such as shrub long axis (L_g_), short axis (W_g_), and windward slope length (L_y_). (2) The surface sediments of nabkhas were mainly composed of silt and fine sand, followed by very fine sand. Compared with the CK, the silt content was generally lower in the shrub-covered plots, whereas the contents of fine sand and very fine sand were higher. The mean grain size (Mz, Φ value) tended to decrease, while the skewness (SK_G_) and kurtosis (K_G_) tended to increase, and the sorting coefficient (σ_G_) showed relatively limited variation. (3) In the LCP, MCP, and HCP, the fractal dimension (D) was significantly positively correlated with the Mz and σ_G_ (*p* < 0.05), and significantly negatively correlated with the SK_G_ and K_G_ (*p* < 0.01), suggesting that the D may be associated with variations in sediment grain size structure. (4) Overall, the nabkhas around *Caragana tibetica* shrubs exhibited a spatial distribution pattern characterized by aggregation at small scales and randomness at large scales, with small-scale clustering being more evident in the MCP and HCP. In general, nabkhas around *Caragana tibetica* shrubs under different vegetation cover conditions showed observable differences in morphological characteristics, surface sediment grain size composition, and spatial distribution patterns, providing a comparative case reference for the study of nabkhas in desert steppe areas.

## 1. Introduction

In arid and semi-arid regions, vegetation is not only an important component of terrestrial ecosystems but also represents an important biotic factor associated with near-surface aeolian sediment transport, particle deposition, and landform evolution. By weakening the near-surface airflow, reducing wind velocity, and intercepting transported sand particles, shrubs are often associated with the formation of nabkhas around their bases, which represent a typical type of biogenic aeolian landform [1,2]. Therefore, nabkhas are not only a direct manifestation of the interaction between aeolian activity and vegetation but also serve as useful indicators for exploring vegetation-controlled sedimentary processes, evaluating the windbreak and sand-fixing functions of shrubs, and assessing surface ecological stability in arid regions [3,4]. From the perspective of ecogeomorphology, the formation and development of nabkhas are generally understood to be influenced by the combined effects of the vegetation’s structural characteristics, the aeolian dynamic conditions, and the sedimentary processes.

In recent years, studies on nabkhas at home and abroad have mainly focused on morphological characteristics, sediment composition and grain size distribution, as well as spatial distribution patterns. Existing studies have shown that vegetation attributes, such as shrub height, crown width, branch structure, and windward characteristics, are associated with the length, height, slope structure, and overall morphological development of nabkhas [5]. The grain size composition of nabkha sediments can provide information about the local aeolian dynamic environment, particle transport modes, and depositional sorting processes, and thus has been widely used as an indicator for identifying changes in the sedimentary environment and the intensity of aeolian activity [6]. On this basis, fractal theory has gradually been introduced into sediment grain size studies, and fractal dimension can be used to describe, to a certain extent, the complexity and variation in the sediment particle structure [7]. In addition, spatial point pattern analysis has been widely applied in studies of plant population distribution and ecological adaptation. nabkhas in different regions may exhibit clustered, random, or uniform spatial structures, and these pattern characteristics have been reported to be related to local environmental heterogeneity, microtopographic variation, and resource allocation processes [8].

Although previous studies have provided an important foundation for understanding the formation and development of nabkhas, most research has focused on typical desert shrubs, such as *Tamarix chinensis* and *Nitraria tangutorum*, whereas systematic studies on the nabkhas associated with dominant shrubs in desert steppe regions remain relatively limited. Compared with typical deserts, desert steppe represents a transitional zone between the steppe and desert, where vegetation cover, aeolian activity, and depositional environments exhibit strong spatial heterogeneity and transitional characteristics. Against this background, differences may be observed in surface sedimentary environments and microtopographic development among plots with different shrub cover. *Caragana tibetica* is an important dominant sand-fixing shrub in desert steppe regions. Its dense branches and well-developed crown are generally considered to contribute to a reduction in wind erosion and promote particle deposition, often leading to the formation of relatively typical nabkhas around the shrub. However, studies on nabkhas associated with *Caragana tibetica* are still scarce, especially comprehensive comparative studies conducted within the same study area and under the same dominant shrub background, integrating nabkha morphological characteristics, surface sediment features, and spatial patterns.

Accordingly, this study takes the nabkhas associated with *Caragana tibetica* in the desert steppe of Damao Banner, Inner Mongolia, as the research object. Under the background of plots with different shrub cover, a comprehensive analysis was conducted from three aspects: nabkha morphological parameters, surface sediment grain size characteristics, and spatial distribution patterns. Compared with previous studies, the emphasis of this research is not on supplementing the description of a single indicator, but rather on incorporating morphology, sediment, and spatial pattern into a unified analytical framework, so as to compare their variation characteristics under consistent site conditions and provide a regional case for understanding vegetation–wind–sediment interactions in desert steppe regions. Specifically, this study addresses the following questions: (1) What differences exist in the morphological characteristics of nabkhas among plots with different shrub covers? (2) How do the grain size composition and fractal characteristics of surface sediments vary among plots with different shrub covers? (3) What scale-dependent characteristics are exhibited by the spatial distribution patterns of nabkhas under different shrub cover conditions?

## 2. Materials and Methods

### 2.1. Study Area

The study area is located in Baiyanhua Gacha, Bailingmiao Town, Damao Banner, Baotou City, Inner Mongolia Autonomous Region (41°57′~42°01′ N, 110°06′~110°24′ E), with an average elevation of 1348 m. The region is characterized by a typical continental arid to semi-arid climate, with a mean annual temperature of approximately 3.4 °C. The extreme minimum temperature can reach −34.5 °C, while the extreme maximum temperature can reach 37 °C. The mean annual precipitation is 233.70 mm, most of which is concentrated from July to September. Due to low precipitation and high evaporation, the degree of regional aridity is relatively severe. The prevailing winds in the study area are northerly and northwesterly, with relatively high wind intensity. Strong wind events occur approximately 6–7 days per year, with wind force reaching 7 on the Beaufort scale. Sand and dust weather is also frequent, with an average of about 20–25 sandstorm days annually. Soil freezing is pronounced in the winter, and the mean annual frozen soil depth is about 22 cm. The dominant soil type is chestnut soil, which is mainly distributed in relatively flat and low-lying areas and is characterized by a comparatively thick humus layer. The vegetation is mainly composed of drought-tolerant shrubs and herbaceous plants, and the community structure is relatively simple, with herbaceous vegetation generally showing a low and sparse distribution. The dominant shrub species is *Caragana tibetica*, while the main herbaceous plant is *Stipa capillata*. Under the combined effects of strong winds and abundant sand supply, various types, sizes, and shapes of nabkhas have developed in the study area, with *Caragana tibetica* as the dominant shrub species.

### 2.2. Field Survey and Plot Design

In April 2024, a field investigation was conducted in the desert steppe region of Damao Banner, Inner Mongolia. The study area was generally flat, with slopes of all plots being less than 3°, indicating that topographic factors were likely to have a relatively limited influence on the morphological development and depositional processes of nabkhas. A typical area with well-developed *Caragana tibetica* nabkhas was selected, and four plots, each measuring 250 m × 350 m, were established: a control plot without shrub distribution (CK), and three shrub-covered plots representing a low cover plot (LCP), a medium cover plot (MCP), and a high cover plot (HCP), respectively. It should be noted that only one plot was established for each cover type; therefore, this study is based on comparisons among four specific plots rather than a replicated plot-level inference for each cover category. The CK served as a shrub-free reference plot for comparing differences in nabkha-related characteristics under conditions with and without shrub cover, while comparisons among LCP, MCP, and HCP were mainly used to describe variation patterns among shrub-covered plots with different levels of cover. Based on this plot design, the results of this study are mainly intended to reveal differences in nabkha morphology, sediment characteristics, and spatial patterns under different cover backgrounds, rather than to make strict general inferences about cover effects.

Using UAV imagery (DJI Phantom 4 RTK, spatial resolution 5 cm) combined with field investigation, the location and number of all nabkhas within each plot were identified and recorded, yielding a total of 674 nabkhas. Two-dimensional spatial coordinates were extracted using the east–west direction as the X-axis and the north–south direction as the Y-axis for spatial pattern analysis. Based on the regional meteorological data and field observations, the windward and leeward slopes of each nabkha were consistently determined, with the prevailing wind directions being northerly and northwesterly. The field observations showed that all shrubs forming nabkhas in the plots were *Caragana tibetica*, and no other shrub species were found to dominate nabkha formation. Some differences were observed among plots in the number of main stems, branching characteristics, trunk inclination, and evolutionary stages of the *Caragana tibetica* shrubs. Their main morphological and structural characteristics are shown in Table 1.

### 2.3. Measurement of Nabkha Morphological Parameters

In May 2024, measurements of nabkha morphological parameters were taken in the CK and in the shrub-covered plots with different levels of shrub cover (LCP, MCP, and HCP). In the LCP, MCP, and HCP, all intact nabkhas were numbered, and 10 samples were randomly selected from each plot using a random number table for measurement. Because only a small number of scattered nabkhas occurred in the control plot (CK), all of them were measured. In total, 34 nabkha samples were obtained for morphological analysis, including all samples from the CK and the 10 randomly selected samples from each of the other plots. The measured variables included shrub long axis (L_g_), shrub short axis (W_g_), shrub height (H_g_), nabkha long axis (L_d_), nabkha short axis (W_d_), nabkha height (H_d_), windward slope length (L_y_), and leeward slope length (L_b_). The windward and leeward slopes were identified according to the prevailing wind direction in the study area. The length of the windward slope was measured from the slope toe to the crest along the ground surface, and the horizontal distance was recorded by stretching the measuring tape straight. The leeward slope length was measured as the horizontal distance from the crest extending down the leeward side to the slope toe. Because the overall shapes of the *Caragana tibetica* shrubs and nabkhas were approximately elliptical, the horizontal scale (HC), projected basal area of the nabkha (S_d_), nabkha volume (V_d_), and shrub canopy cover area (S_g_) were calculated according to previously reported methods. The equations are as follows [10]:

(1) $$HC=\frac{1}{2}\left(L_{d}+W_{d}\right)$$

(2) $$S_{d}=\frac{1}{2}\left(L_{d}\times W_{d}\right)$$

(3) $$V_{d}=\frac{1}{6}(L_{d}\times W_{d}\times H_{d})$$

(4) $$S_{g}=\frac{1}{2}\left(L_{g}\times W_{g}\right)$$

### 2.4. Grain Size Characteristics of Surface Sediments

#### 2.4.1. Sediment Sampling and Grain Size Analysis

To investigate the surface sediment characteristics of nabkhas under different shrub-cover conditions, three parallel transects were established in each shrub-cover plot using a systematic random sampling design, with a spacing of 50 m between transects. Along each transect, three nabkhas were randomly selected, with a minimum spacing of 10 m between adjacent nabkhas to avoid spatial auto-correlation, resulting in a total of nine nabkhas in each shrub-cover plot. The surface sediment samples were collected from the 0–10 cm layer. A five-point composite sampling method was used. After removing the surface litter, subsamples were thoroughly mixed and sealed in self-locking bags for transport to the laboratory. Because the number of nabkhas in the control plot (CK) was limited, all nabkhas in this plot were sampled. After natural air-drying, the samples were cleaned of impurities, ground, sieved, and sub-sampled. Prior to the particle size analysis, samples were pretreated following the method proposed by Konert et al. [11]. The soil particle size composition was then determined using a Malvern Mastersizer 2000M laser particle size analyzer (Malvern Panalytical, Malvern, UK; measurement range: 0.02–2000 μm). Each sample was measured three times, and the mean value was used when the measurement error was less than 2%. According to the United States Department of Agriculture (USDA) soil particle size classification standard, the soil particles were classified into very coarse sand (1000–2000 μm), coarse sand (500–1000 μm), medium sand (250–500 μm), fine sand (100–250 μm), very fine sand (50–100 μm), silt (2–50 μm), and clay (<2 μm) [12].

#### 2.4.2. Calculation of Grain Size Parameters

The grain size data were logarithmically transformed using the method proposed by Krumbein [13]:

(5) $$\Phi =-\log_{2}d$$

The grain size parameters, including the mean grain size (Mz), sorting coefficient (σ_G_), skewness (SK_G_), and kurtosis (K_G_), were calculated using the Folk–Ward graphical method [14]. These parameters respectively reflect the particle coarseness, the degree of dispersion in particle distribution, the symmetry of the grain size distribution, and the sharpness of the grain size distribution curve (Table 2) [15,16]. The grain size parameters were calculated using GRADISTAT Version 8.0 [17].

#### 2.4.3. Calculation of Fractal Dimension

The fractal dimension of sediments was calculated based on the particle size volume data obtained from the laser particle size analyzer:

(6) $$\frac{V_{\left(r<Ri\right)}}{V_{T}}={\left(\frac{R_{i}}{R_{max}}\right)}^{\left(3-D\right)}$$

where *D* is the fractal dimension of the soil; *r* is the soil particle size (μm); *R_i_* is the particle size of grade *i* (μm); *R_max_* is the maximum soil particle size (μm); *V*(*r* < *R_i_*) is the volume fraction (%) of soil particles with a particle size smaller than *R_i_*; and *V_T_* is the total volume fraction (%) of all particle size classes.

### 2.5. Spatial Point Pattern and Statistical Analysis

The spatial distribution patterns of *Caragana tibetica* nabkhas under different shrub-cover conditions were analyzed using the pair correlation function g(r), which is derived from Ripley’s K function [18]. When the function lies above the upper confidence envelope, the population is considered to exhibit a clustered distribution; when it falls below the lower confidence envelope, it indicates a uniform distribution; and when it lies between the two envelopes, the distribution is considered random [19].

(7) $$K\left(r\right)=\frac{A}{n^{2}}\sum_{i=1}^{n}\sum_{j=1}^{n}\frac{I_{r}\left(u_{ij}\right)}{W_{ij}}\left(i\neq j\right)$$

(8) $$g\left(r\right)={\left(2\pi r\right)}^{-1}\frac{dK\left(r\right)}{dr}$$

where r is the spatial scale; A is the area of the study plot; n is the total number of plant individuals in the plot; and uᵢⱼ is the distance between individuals i and j. When uᵢⱼ ≤ r, then Iᵣ(uᵢⱼ) = 1; when uᵢⱼ > r, then Iᵣ(uᵢⱼ) = 0. Wᵢⱼ represents the ratio of the arc length of the circle (centered at i with radius uᵢⱼ) that lies within the study area A to the circumference of the circle.

The statistical analyses of the soil particle size fractions, particle size parameters, and fractal dimensions were performed using Excel 2016 and SPSS 27.0. The data were first tested for normality using the Shapiro–Wilk test and for homogeneity of variance using Levene’s test. When the assumptions were satisfied, one-way analysis of variance (one-way ANOVA) was used to compare the differences among the plots with different shrub-cover levels, and the F values and their significance levels (*p* values) were reported. When significant differences were detected, Tukey’s honestly significant difference (HSD) test was further applied for multiple comparisons. The correlations among variables were analyzed using the Pearson correlation analysis, and the correlation coefficients(r) and their significance levels were reported. Spatial point pattern analysis was conducted in the spatstat package in R (4.2.2) to examine the spatial distribution characteristics of the sampling points, with the aim of describing spatial pattern differences among plots under the present sampling design.

## 3. Results and Analysis

### 3.1. Morphological Characteristics of Nabkhas Under Different Shrub-Cover Plots

The *Caragana tibetica* nabkhas in Baiyanhua Gacha, Damao Banner, were mostly ellipsoidal or semi-ellipsoidal in shape, with relatively gentle slopes. As shown in Table 3, some morphological parameters of nabkhas varied among the plots with different shrub-cover levels, although the responses of the different parameters were not entirely consistent. The shrub long axis (L_g_) of nabkhas in the MCP was 77.00 cm, which was significantly greater than that in the LCP (67.40 cm), HCP (63.60 cm), and CK (64.80 cm) (*p* < 0.05). For the shrub short axis (W_g_), the value in the MCP was 68.90 cm, which was significantly higher than that in the CK (62.20 cm) (*p* < 0.05), but not significantly different from those in the LCP (67.10 cm) and HCP (63.50 cm) (*p* > 0.05). Significant differences were also found in the windward slope length (L_y_) among plots: the MCP (42.20 cm) and LCP (39.20 cm) both had significantly greater values than the HCP (30.50 cm) (*p* < 0.05). In contrast, the shrub height (H_g_), nabkha long axis (L_d_), nabkha short axis (W_d_), nabkha height (H_d_), and leeward slope length (L_b_) did not differ significantly among the shrub-cover plots (*p* > 0.05). Overall, the MCP exhibited relatively higher values for several parameters, including L_g_, W_g_, and L_y_, although this trend was not consistently observed across all morphological parameters.

**Figure 1:**
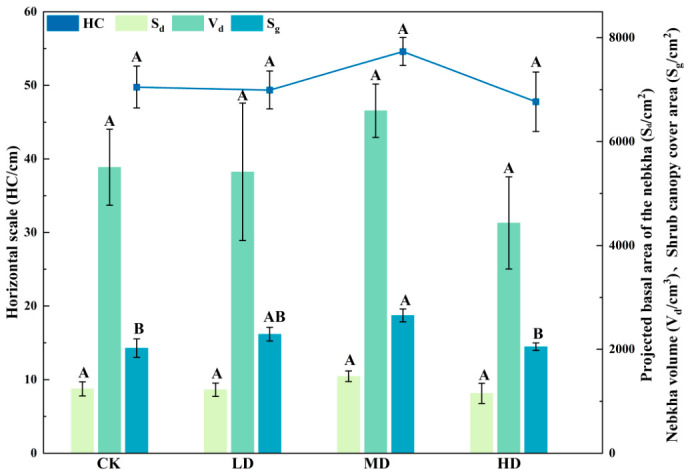
The scale characteristics of nabkhas under different shrub-cover plots. Note: CK, LCP, MCP, and HCP represent the control plot, low-cover plot, medium-cover plot, and high-cover plot, respectively. HC denotes horizontal scale, S_d_ denotes the projected basal area of the nabkha, V_d_ denotes the nabkha volume, and S_g_ denotes the shrub canopy cover area. The bars indicate the mean values ± standard error. The different uppercase letters indicate significant differences among the plots with different shrub-cover levels (*p* < 0.05).

As shown in Figure 1, the horizontal scale (HC), projected basal area of the nabkha (S_d_), nabkha volume (V_d_), and shrub canopy cover area (S_g_) of *Caragana tibetica* nabkhas showed some variation among plots with different shrub-cover levels, although not all indices differed significantly. The HC did not differ significantly among the plots (*p* > 0.05), ranging from 47 to 55 cm overall, with slightly higher values in the MCP and relatively lower values in the HCP. The S_d_ also showed no significant differences among the shrub-cover plots (*p* > 0.05), although the MCP showed slightly higher values than the other plots. Similarly, V_d_ did not differ significantly among the plots (*p* > 0.05), but followed the trend of MCP > CK > LCP > HCP overall. S_g_ differed to some extent among the plots, with the MCP showing significantly higher values than the CK and HCP (*p* < 0.05), while no significant difference was found between the MCP and LCP (*p* > 0.05). Overall, the MCP exhibited relatively higher values for some indices, but this was not sufficient to indicate a consistent advantage across all morphological development parameters.

### 3.2. Correlations Between Shrub and Nabkha Morphological Parameters Under Different Shrub-Cover Plots

As shown in Table 4, the correlations between shrub and nabkha morphological parameters varied among plots with different shrub-cover levels. In the CK, all the parameter pairs showed highly significant correlations (*p* < 0.01), except for the relationship between H_g_ and H_d_, which was not significant (*p* > 0.05). In particular, L_g_ and W_g_ were both significantly and positively correlated with all the nabkha morphological parameters. In the LCP, the correlations between the shrub and nabkha morphological parameters were not significant (*p* > 0.05). However, the relationships between the nabkha morphological parameters and the nabkha scale characteristics (HC, S_d_, and V_d_) were generally highly significant (*p* < 0.01). In the MCP, the correlations between the shrub morphological parameters and the nabkha morphological parameters were generally stronger than those observed in the LCP. Both L_g_ and W_g_ were highly significantly correlated with W_d_ (*p* < 0.01), and the shrub morphological parameters also showed significant correlations with the nabkha scale characteristics (*p* < 0.05). In addition, W_d_ was highly significantly correlated with S_d_ (r = 0.90, *p* < 0.01), while the HC showed the strongest correlation with L_d_ (r = 0.98, *p* < 0.01). In the HCP, H_g_ was not significantly correlated with H_d_, HC, S_d_, and V_d_ (*p* > 0.05), whereas the remaining parameter pairs still showed highly significant correlations (*p* < 0.01). Meanwhile, the correlation coefficient between V_d_ and H_d_ increased from 0.36 in the MCP to 0.82 in the HCP, showing a stronger statistical association between these two variables in the HCP.

### 3.3. Grain Size of Surface Sediments in Nabkha Dunes Under Shrub Stands Across Sample Plots with Different Coverage Levels

**Figure 2:**
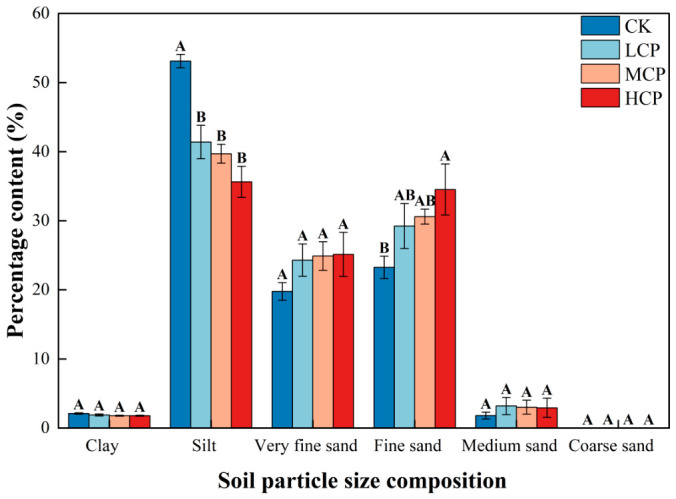
The particle composition of surface sediments in nabkha dunes under shrub stands across sample plots with different coverage levels. The different uppercase letters indicate significant differences among the plots with different shrub-cover levels (*p* < 0.05).

As shown in Figure 2, the surface sediments of the nabkha dunes were mainly composed of silt and fine sand, followed by very fine sand, whereas the contents of clay, medium sand, and coarse sand were relatively low. Among the sample plots with different coverage levels, the contents of clay and coarse sand were low and showed no significant differences (*p* > 0.05). The silt content in the CK was significantly higher than that in the other coverage plots (*p* < 0.05), while no significant differences were found among the LCP, MCP, and HCP (*p* > 0.05). The content of fine sand generally increased across the plotted coverage gradient, reaching the highest value in the HCP, which was significantly higher than that in the CK (*p* < 0.05). The content of very fine sand did not differ significantly among the different coverage plots (*p* > 0.05), but the values in the LCP, MCP, and HCP were all higher than those in the CK. Overall, compared with the CK, the surface sediments of nabkha dunes in the different coverage plots were characterized by a lower proportion of silt and higher proportions of fine sand and very fine sand.

**Figure 3:**
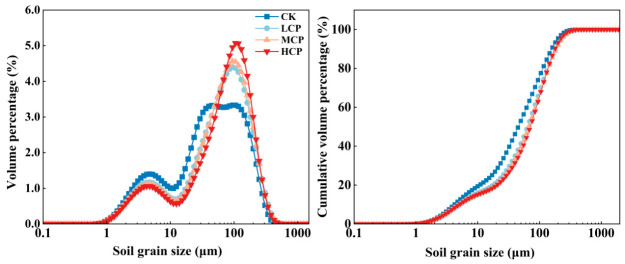
The grain size frequency distribution curves and cumulative frequency curves of surface sediments in nabkha dunes under shrub stands across sample plots with different coverage levels.

As shown in Figure 3, the grain size frequency distribution curves of sediments under different coverage plots all exhibited a bimodal pattern. The first peak was mainly located around 1–10 μm, whereas the second peak occurred at approximately 50–100 μm. Differences were observed in the relative height of the second peak among the different coverage plots. Specifically, the HCP showed the highest volumetric percentage near the second peak, whereas the CK showed the lowest, indicating differences in the distribution structure of the dominant grain size fractions among the plots. The cumulative frequency curves showed that sediments in all coverage plots were mainly concentrated within the 10–200 μm range. Compared with the CK, the cumulative curves of the LCP, MCP, and HCP rose more steeply within this grain size range, indicating that their grain size distributions were relatively more concentrated. Overall, the surface sediments of nabkha dunes under different coverage plots all displayed a bimodal characteristic, but differences existed in the peak intensity and in the degree of concentration within the main grain size range.

**Figure 4:**
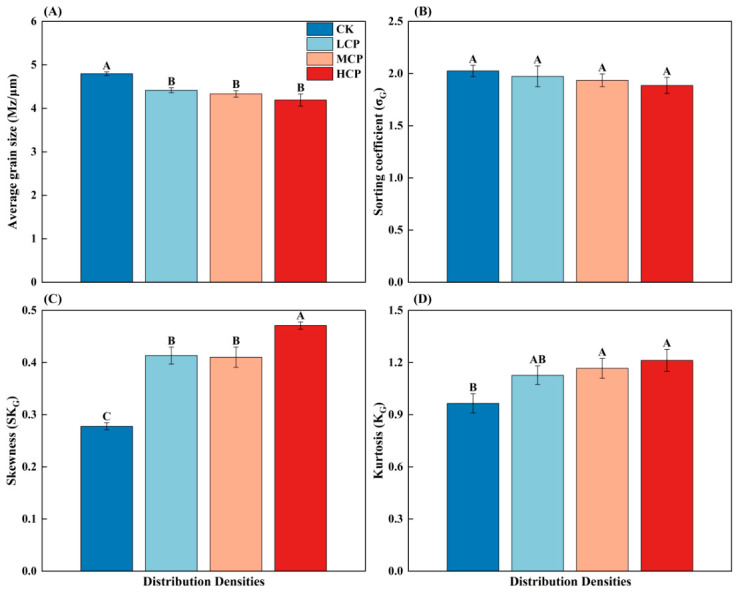
Effects of different vegetation patch distribution densities on soil particle size distribution characteristics (**A**) Average grain size (Mz, μm); (**B**) Sorting coefficient (σ_G_); (**C**) Skewness (SK_G_); (**D**) Kurtosis (K_G_). The different uppercase letters indicate significant differences among the plots with different shrub-cover levels (*p* < 0.05). Error bars represent the standard error of the mean (n = 3).

As shown in Figure 4, there were certain differences in the grain size parameters of surface sediments among the sample plots with different coverage levels. The CK had the largest mean grain size (4.79 Φ), which was significantly higher than that of the other coverage plots (*p* < 0.05). The mean grain sizes (Mz) of the LCP, MCP, and HCP were 4.41Φ, 4.33Φ, and 4.19Φ, respectively, with no significant differences among them (*p* > 0.05). The sorting coefficient (σ_G_) showed no significant difference among the different coverage plots (*p* > 0.05), and its overall variation was relatively small. In contrast, the skewness (SK_G_) and kurtosis (K_G_) showed more obvious changes. The SK_G_ of the CK was the lowest (0.28), significantly lower than that of the other coverage plots (*p* < 0.05), whereas the HCP had the highest SK_G_ (0.47), significantly higher than that of the other coverage plots (*p* < 0.05). K_G_ was the lowest in the CK (0.96), while relatively higher values were observed in the MCP and HCP. Overall, the variation in sediment grain size parameters among the different coverage plots was mainly characterized by a decrease in mean grain size (Φ value) and increases in the SK_G_ and K_G_, whereas the σ_G_ changed only slightly. These results indicate differences in the grain size structure among plots, although not all parameters responded in the same way.

**Figure 5:**
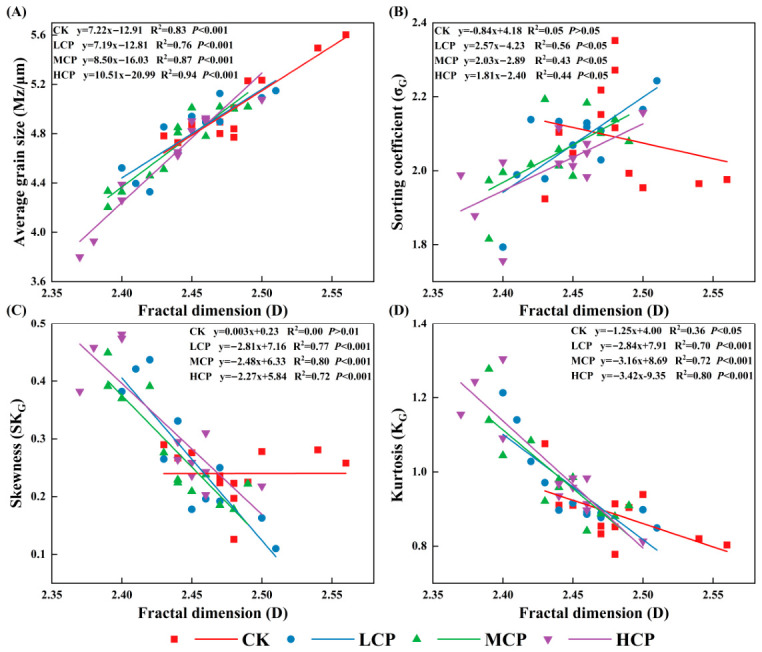
Relationships between fractal dimension (D) and grain-size parameters under different coverage plots (CK, LCP, MCP, and HCP). (**A**) Relationship between fractal dimension (D) and average grain size (Mz, μm). (**B**) Relationship between fractal dimension (D) and sorting coefficient (σ_G_). (**C**) Relationship between fractal dimension (D) and skewness (SK_G_). (**D**) Relationship between fractal dimension (D) and kurtosis (K_G_). Symbols represent different treatments: CK (red squares), LCP (blue circles), MCP (green triangles), and HCP (purple inverted triangles); lines indicate fitted linear regressions.

Scatter plots were established between the grain size parameters and the fractal dimension (D) for the CK, LCP, MCP, and HCP, respectively (Figure 5), in order to analyze the relationships between the fractal dimension and grain size parameters under different coverage conditions. As shown in Figure 5A, the Mz was significantly and positively correlated with a fractal dimension in all coverage plots, with relatively strong correlations (CK: R^2^ = 0.83, LD: R^2^ = 0.76, MD: R^2^ = 0.87, HD: R^2^ = 0.94; all *p* < 0.001). This indicates that, within each coverage plot, the value of the Mz increased with increasing D. As shown in Figure 5B, the relationship between the σ_G_ and the D differed among the coverage plots. In the CK, the correlation was not significant (R^2^ = 0.05, *p* > 0.05), whereas significant positive correlations were observed in the LCP, MCP, and HCP (LD: R^2^ = 0.56, MD: R^2^ = 0.43, HD: R^2^ = 0.44; all *p* < 0.05). This pattern indicates a positive association between the D and the σ_G_ in these shrub-covered plots. As shown in Figure 5C, the SK_G_ was not significantly correlated with the D in the CK (R^2^ = 0.00, *p* > 0.05) but showed highly significant negative correlations in the LCP, MCP, and HCP (LD: R^2^ = 0.77, MD: R^2^ = 0.80, HD: R^2^ = 0.72; all *p* < 0.001), showing that the SKG generally decreased as the D increased in these plots. As shown in Figure 5D, K_G_ was negatively correlated with the D in all plots. The correlation was significant in the CK (R^2^ = 0.36, *p* < 0.05) and highly significant in the LCP, MCP, and HCP (LD: R^2^ = 0.70, MD: R^2^ = 0.72, HD: R^2^ = 0.80; all *p* < 0.001), showing that K_G_ generally decreased as the D increased. Overall, the D showed relatively stable relationships with the Mz, σ_G_, SK_G_ and K_G_, with more pronounced correlations in the LCP, MCP, and HCP.

### 3.4. Spatial Distribution Patterns of Nabkha Dunes Under Different Shrub Coverage Plots

As shown in Table 5, there were clear differences in the number of nabkha dunes and their spatial coverage among the sample plots with different coverage levels. The CK had the smallest number of nabkha dunes, with 48 dunes recorded. In comparison, the LCP, MCP, and HCP contained 129, 209, and 376 nabkha dunes, respectively. At the same time, the proportion of the sample plot area occupied by the vertical projection area of nabkha dunes increased from 18.30% in the CK to 38.80% in the LCP, 57.20% in the MCP, and 78.50% in the HCP. Overall, obvious differences were found in both the number of nabkha dunes and their spatial coverage proportion among the different coverage plots, with the HCP showing the highest dune number and coverage proportion.

**Figure 6:**
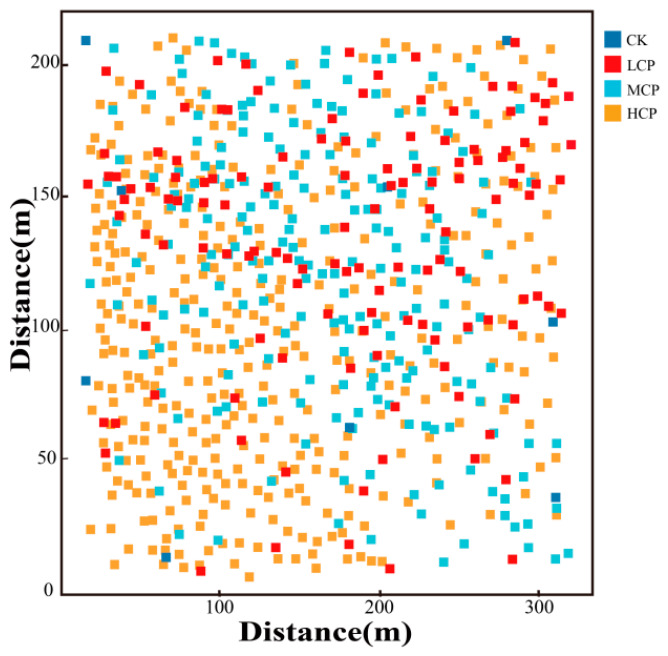
The spatial distribution of nabkhas dunes under different coverage plots.

As shown in the two-dimensional spatial distribution maps of nabkha dunes in Figure 6, the nabkha dunes in the different coverage plots did not exhibit obvious regular distribution patterns, although differences were observed in the degree of spatial aggregation and dispersion. The HCP contained the largest number of nabkha dunes, which were distributed relatively densely across the plot and formed obvious patchy patterns in some local areas. In the MCP, the nabkha dunes were distributed relatively evenly, although a certain degree of local aggregation was still present. In the LCP, the number of nabkha dunes was relatively small, and their overall distribution was comparatively scattered. The CK had the fewest nabkha dunes, and their overall distribution was more dispersed. Overall, nabkha dunes in all coverage plots showed spatial characteristics in which local aggregation and random distribution coexisted.

**Figure 7:**
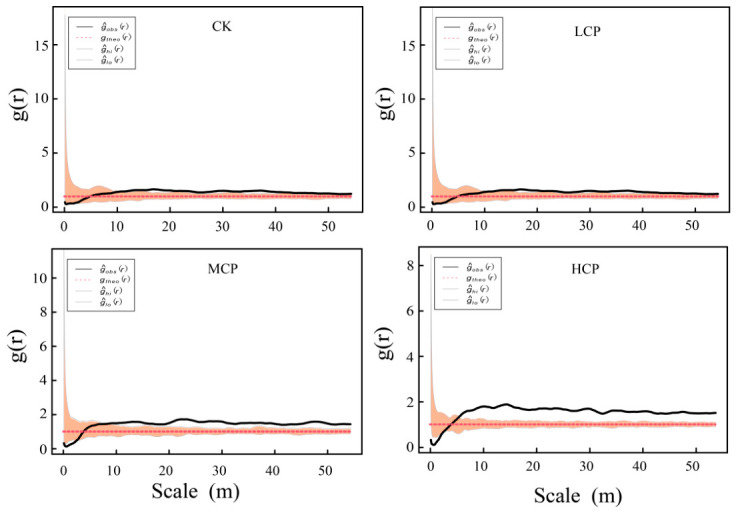
The spatial pattern characteristics of nabkhas dunes under different coverage plots.

To further analyze the spatial distribution characteristics of shrub–sand mounds at different scales using the paired correlation function g(r) (Figure 7). The results showed that at small scales (approximately 0–10 m), the observed function values gobs(r) for all cover plots were generally higher than the theoretical random distribution curve gtheo(r), indicating a tendency toward clustered distribution at this scale. As the spatial scale increased, gobs(r) gradually approached the theoretical random distribution curve and stabilized, indicating that the distribution of sand mounds gradually became more random at larger scales. When comparing different cover plots, the MCP and HCPs exhibited relatively higher g(r) values at small scales, with a more pronounced degree of clustering; the CK and LCP showed a relatively weaker degree of clustering but still exhibited small-scale clustering characteristics overall. Beyond approximately 10 m, the g(r) curves for all coverage plots gradually stabilized and largely fell within the confidence interval of a random distribution, indicating that the spatial distribution of shrub–sand mounds approached randomness on medium and large scales. Overall, the *Caragana tibetica* nabkhas in the study area showed a spatial pattern characterized by “small-scale aggregation and large-scale randomness,” with differences among coverage plots primarily reflected in the degree of small-scale aggregation.

## 4. Discussion

### 4.1. Morphological Differences in Nabkhas Among Plots with Different Shrub Cover

The morphological characteristics of nabkhas are an important manifestation of the interaction between vegetation and aeolian processes. To some extent, their morphological parameters can be used to describe local depositional environments and vegetation-induced sand trapping and are, therefore, commonly used to characterize nabkha development. Previous studies have shown that nabkha morphology is jointly influenced by multiple factors, including vegetation structure, wind–sand dynamics, and sedimentary processes, and that their morphological parameters often differ markedly among vegetation types and habitat settings [20]. The results of this study showed that the morphological parameters of *Caragana tibetica* nabkhas varied among the plots with different shrub-cover levels. In particular, the MCP exhibited relatively high values for L_g_, W_g_, and L_y_, indicating the relatively greater values of these parameters in this plot.

Further correlation analysis between the shrub and nabkha morphological parameters showed that, in the CK and the MCP, the L_g_ and W_g_ were generally significantly correlated with nabkha morphological parameters, suggesting that shrub morphology was statistically associated with nabkha morphology in these plots. In contrast, these correlations were relatively weaker in the LCP, indicating that the response of the nabkha morphology to the shrub structure was not consistent among plots with different coverage levels. Similar patterns have also been reported in studies of other nabkha-forming shrubs, such as *Nitraria tangutorum* [21], suggesting that nabkha morphology is often associated with the joint effects of vegetation structure and local environmental conditions, rather than being determined by a single factor alone.

Regarding the mechanisms underlying these differences, we suggest that they may be related to differences in the local aeolian sedimentary conditions among plots with different shrub-cover levels. The generally larger morphological parameters of nabkhas in the MCP are consistent with the possibility that moderate shrub cover may be more favorable for particle deposition around shrubs and for nabkha preservation. In contrast, the reduction in some morphological indices in the HCP may be associated with interference from neighboring shrubs or with spatial competition under the HCP. Previous studies have shown that shrub population structure can influence depositional processes around individual shrubs by altering the near-surface airflow conditions and particle transport pathways [22,23,24]. Therefore, the morphological differences observed among the different shrub-cover plots in this study may be interpreted as plot-level differences associated with the local depositional environment under different shrub-cover backgrounds. However, it should be noted that near-surface wind speed, airflow structure, and sediment transport processes were not directly measured in this study. Therefore, interpretations concerning airflow regulation, optimized particle deposition, and changes in local wind fields remain speculative, based on morphological differences and the previous literature, and still require further verification through wind tunnel experiments, field wind-speed monitoring, or sediment transport observations.

In addition, compared with the previous studies on *Caragana tibetica* nabkhas in Western Ordos, the nabkhas in the present study area were generally smaller in size [25,26]. This difference may be related to the regional environmental conditions, such as precipitation, wind erosion intensity, and surface material supply, although the data available in this study are insufficient to identify the dominant mechanism with certainty. It should also be noted that the plots in this study were generally flat, with slopes of less than 3°, and the influence of microtopographic relief on nabkha morphological development was, therefore, relatively limited. For this reason, the slope was not included as an independent variable in the analysis. Overall, the results indicate that the morphological development of *Caragana tibetica* nabkhas differed markedly among plots with different shrub-cover levels, although the process-based mechanisms underlying these differences still require further testing through direct observations of aeolian dynamics.

### 4.2. Differences in Sediment Grain Size Characteristics Among Plots with Different Shrub Cover

Sediment grain size characteristics are important indicators of aeolian depositional environments and their dynamic conditions and are commonly used in aeolian geomorphology to analyze sediment sources and transport modes. It should be noted that the present study mainly compared depositional responses among plots with different shrub-cover levels based on the surface sediment grain size composition, grain size parameters, and fractal characteristics, without directly observing near-surface wind fields or sediment transport processes. Therefore, the following discussion is primarily an interpretive analysis based on the observed results and previous studies, rather than direct evidence of the specific dynamic processes. The results showed that the surface sediments of *Caragana tibetica* nabkhas under different shrub-cover conditions were generally dominated by silt and fine sand, followed by very fine sand, whereas clay, medium sand, and coarse sand were present in relatively low proportions. This is broadly consistent with previous findings on the grain size composition of aeolian sediments in the desert steppe regions [27]. At the same time, the grain size frequency distribution curves were generally bimodal, with the main peaks concentrated in the particle size ranges of approximately 1–10 μm and 50–100 μm, indicating that the sediment grain size structure under different shrub-cover conditions displayed a clear bimodal pattern. Combined with previous studies, this bimodal structure may be related to the input and transport modes of particles of different size fractions [28,29]. However, for the present study, a more cautious conclusion is that the surface sediments of nabkhas under different shrub-cover conditions showed plot-level differences in their dominant grain size composition and distribution structure.

A comparison among the different plots showed that, with increasing shrub cover, the sediment grain size composition was generally characterized by a decrease in silt content and increases in fine sand and very fine sand contents. The analysis of grain size parameters further showed that the Φ value of the Mz generally decreased, whereas the SK_G_ and K_G_ increased overall, while the sorting coefficient changed only slightly. It should be noted that the Mz was expressed on the Φ scale, and a decrease in its value corresponds to a slight coarsening of the overall sediment grain size. This is consistent with the observed decrease in silt content and increase in the proportions of fine sand and very fine sand. Therefore, the pattern revealed in this study does not represent a simple process of “continuous increase in fine particles”; rather, it reflects an adjustment in the sediment grain size composition and distribution structure among the plots with different shrub-cover levels, in which the proportion of finer silt decreased, while the relative proportions of fine sand and very fine sand increased within the overall sediment structure.

Accordingly, the differences among plots with different shrub-cover levels are more appropriately interpreted as changes in sediment grain size structure and sorting outcomes, rather than being directly attributed to a single dynamic aeolian process. In other words, what this study can demonstrate is that changes in shrub-cover background correspond to variations in the sediment grain size composition, frequency-curve shape, and grain size parameters. However, whether these relationships were caused by changes in near-surface wind fields, differences in particle transport, or the combined effects of other local environmental factors still requires further verification through direct observations of wind speed, sediment transport flux, and depositional processes. Although previous studies have suggested that higher vegetation cover may influence particle deposition by altering the local wind-field conditions [30], the present study did not directly test this mechanism, and such interpretations should, therefore, remain cautious.

The fractal dimension (D) can be used to characterize, to some extent, the complexity of sediment particle structure [31]. The results of this study showed that fractal dimension (D) was significantly correlated with mean grain size, sorting coefficient, skewness, and kurtosis. Specifically, the D was positively correlated with the mean grain size and sorting coefficient, but negatively correlated with skewness and kurtosis. This indicates that, within each coverage plot, changes in fractal dimension had relatively stable relationships with variations in grain size parameters. However, it should be emphasized that these correlations reflect coordinated changes among grain size structural variables within the individual plots and are not at the same analytical level as the between-plot differences discussed above. In other words, the positive correlation between the fractal dimension and the mean grain size does not mean that the grain size composition changes among different shrub-cover plots can be simply summarized as a single process of “enhanced fine-particle deposition.” Rather, it suggests that the fractal dimension reflects adjustments in the overall structure of grain size distribution, rather than merely the increase or decrease in a particular particle size fraction. Previous studies have also pointed out that the fractal dimension can, to some extent, indicate changes in depositional environments [32]. Therefore, the coordinated changes between the fractal dimension and the grain size parameters observed in this study suggest differences in the structural organization of the grain size among plots, although the specific mechanisms still need to be verified through observations of wind fields and sediment transport processes.

### 4.3. Spatial Distribution Pattern Characteristics of Nabkhas Among Plots with Different Shrub Cover

The spatial distribution patterns are an important means of characterizing the spatial organization of individual shrubs or shrub patches within a plot, and can be used to identify clustered, random, or regular distribution states at different scales [33]. Based on the two-dimensional spatial distribution maps and the multiscale analysis of the pair correlation function g(r), this study showed that the *Caragana tibetica* nabkhas in the study area generally exhibited a spatial distribution pattern characterized by small-scale clustering and large-scale randomness. This pattern was generally consistent among plots with different shrub-cover levels, although the degree of clustering varied to some extent, with more pronounced small-scale clustering in the MCP and HCP. At the same time, clear differences were found in the spatial organization characteristics of nabkhas among plots with different shrub-cover levels, indicating that the spatial organization of nabkhas was not entirely the same under different shrub-cover conditions.

In arid and semi-arid ecosystems, small-scale clustered distribution is commonly considered to be related to resource limitation, habitat heterogeneity, and the establishment processes of plant individuals [9,24,34]. In the present study, the small-scale clustering of the *Caragana tibetica* nabkhas may be associated with the local concentration of shrub individuals and the similarity of the depositional environments surrounding them. In contrast, the tendency toward randomness at larger scales may reflect the absence of a stronger regular spatial structure in shrub distribution and nabkha development at the plot scale [35]. It should be noted that near-surface wind fields, sediment transport processes, soil moisture, and nutrient conditions were not directly measured in this study. Therefore, the above interpretations remain speculative, based on the observed spatial patterns and previous studies.

Previous studies have shown that clustered patterns of vegetation patches may be associated with changes in the local depositional environments, resource redistribution, and vegetation regeneration processes [36,37]. Therefore, the “small-scale clustering and large-scale randomness observed” for the *Caragana tibetica* nabkhas in this study may be interpreted as being consistent with local environmental heterogeneity in desert steppe regions. However, the specific relationships between this spatial pattern and the aeolian depositional processes, resource conditions, and vegetation growth still need to be further verified through direct observations of wind fields, sediment transport, and soil environmental conditions.

## 5. Conclusions

This study focused on *Caragana tibetica* nabkhas in the desert steppe of Damao Banner, Inner Mongolia. Using field surveys, UAV-based image interpretation, grain size analysis, and spatial point pattern analysis, we comparatively analyzed the morphological characteristics, surface sediment grain size composition, and spatial distribution patterns of nabkhas in a shrub-free reference plot (CK) and in shrub-covered plots with a low, a medium, and a high cover (LCP, MCP, and HCP). Because only one plot was established for each shrub-cover category, the results should be interpreted as comparative differences among these specific plots rather than replicated statistical inferences of the shrub-cover effects. The main conclusions are as follows:

(1) Under different shrub-cover conditions, some morphological parameters of *Caragana tibetica* nabkhas varied among plots, although the responses of individual indices were not entirely consistent. In particular, the L_g_, W_g_, and L_y_ were relatively greater in the MCP, whereas H_g_, L_d_, W_d_, H_d_, and L_b_ did not differ significantly among plots.
(2) The surface sediments of the *Caragana tibetica* nabkhas were mainly composed of silt and fine sand, followed by very fine sand. Compared with the control plot, the shrub-covered plots generally showed lower silt contents and higher fine sand and very fine sand contents. The grain size parameter analysis further showed that the Φ value of the Mz generally decreased, while the SK_G_ and K_G_ increased overall, whereas the sorting coefficient varied only slightly. These results indicate differences in the grain size structure among plots with different shrub cover.
(3) The D showed relatively stable relationships with the grain size parameters. In the LCP, MCP, and HCP, the D was significantly positively correlated with the Mz and σ_G_, but significantly or highly significantly negatively correlated with the SK_G_ and K_G_. These relationships reflect a coordinated variation among grain size structural parameters within the plots.
(4) Overall, the *Caragana tibetica* nabkhas exhibited a spatial distribution pattern characterized by “small-scale clustering and large-scale randomness”. The marked differences were observed among the plots with different shrub-cover levels in terms of nabkha number and spatial coverage proportion, with relatively stronger small-scale clustering in the MCP and HCP. These differences highlight a variation in the spatial organization among the plots under different shrub-cover backgrounds.

## Figures and Tables

**Table 1:** The morphological and structural characteristics of the *Caragana tibetica* shrubs under different shrub-cover plots.

Type	UAV Aerial Image	Typical Shrub Photo	Number of Main Stems	Branching Characteristics	Main Stem Inclination	Shrub Evolution Stage
Control plots (CK)	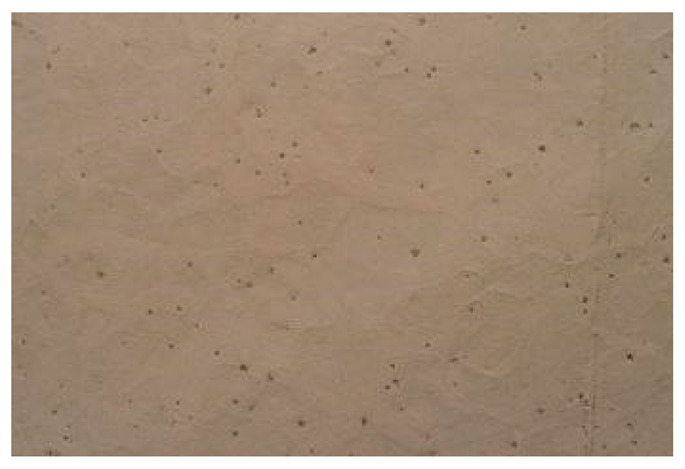	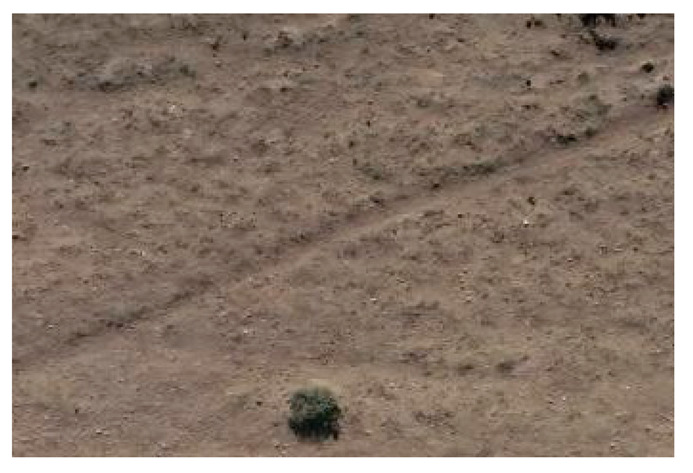	1–3	Few branches and a simple structure	Mostly upright	Mainly in the development stage
Low-cover plot (LCP)	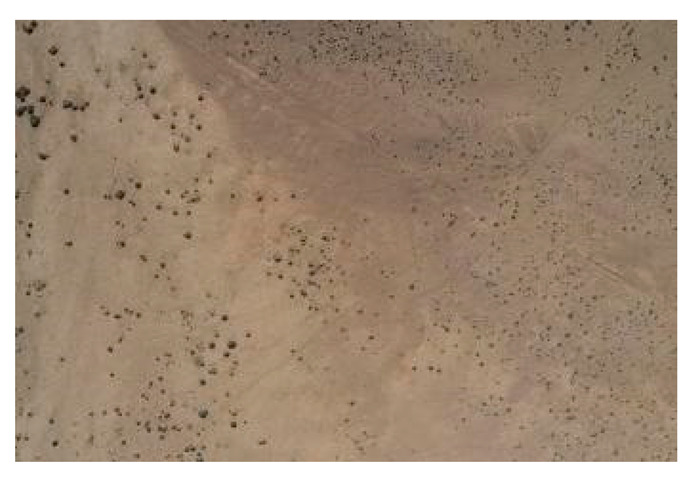	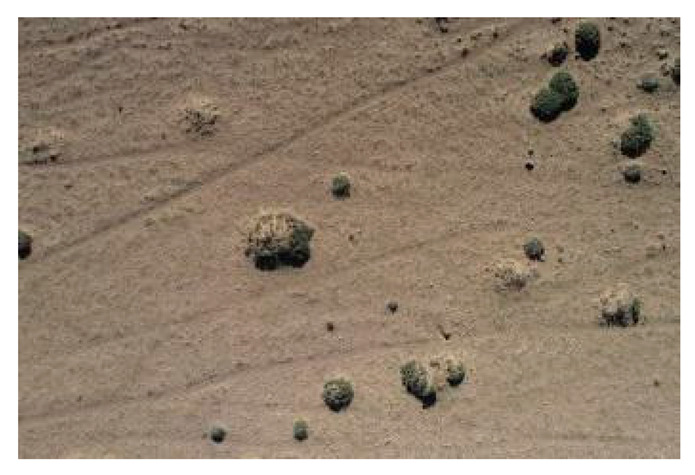	6–12	Branches are relatively dispersed, and the crown shape is intact	Some main stems slightly inclined	Mainly in the development and stable stages
Medium-cover plot (MCP)	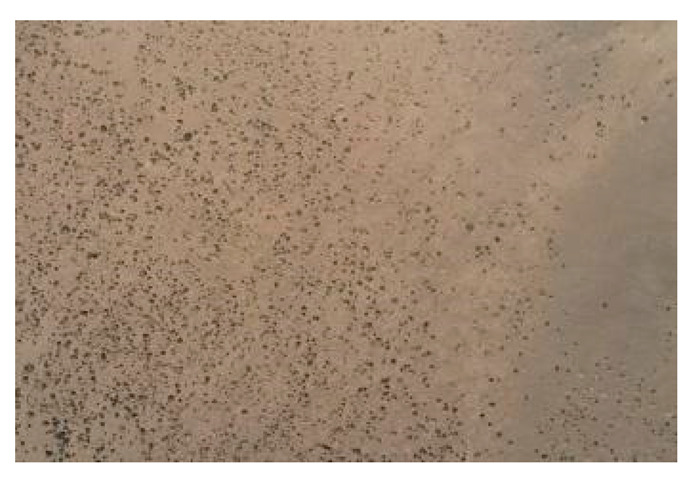	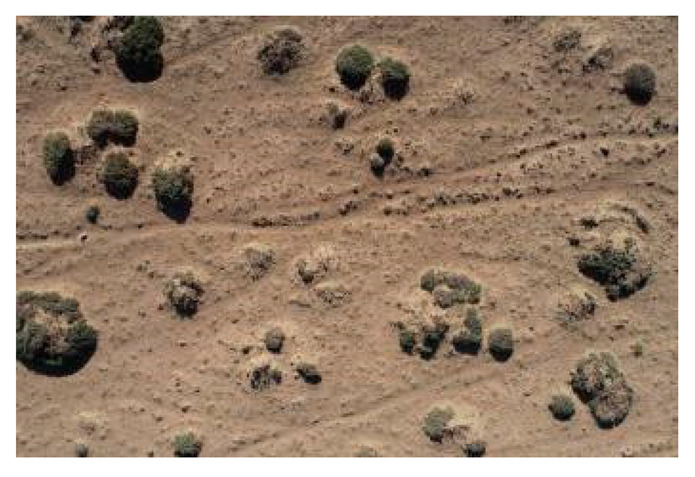	4–8	Dense branching, which is locally interlaced	Main stems mostly upright	Mainly in the stable stage
High-cover plot (HCP)	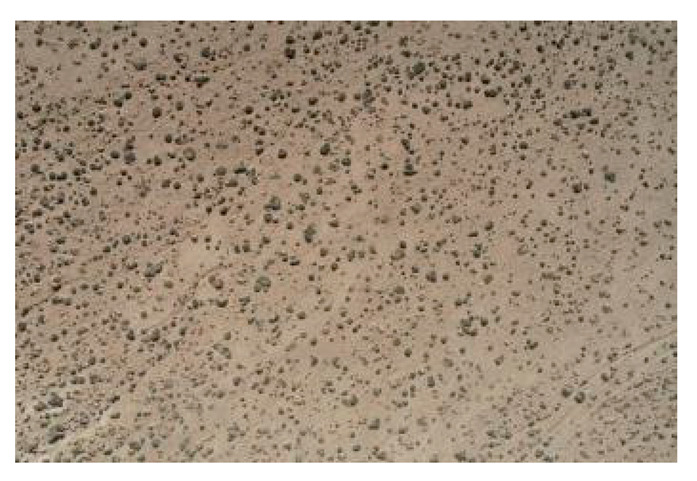	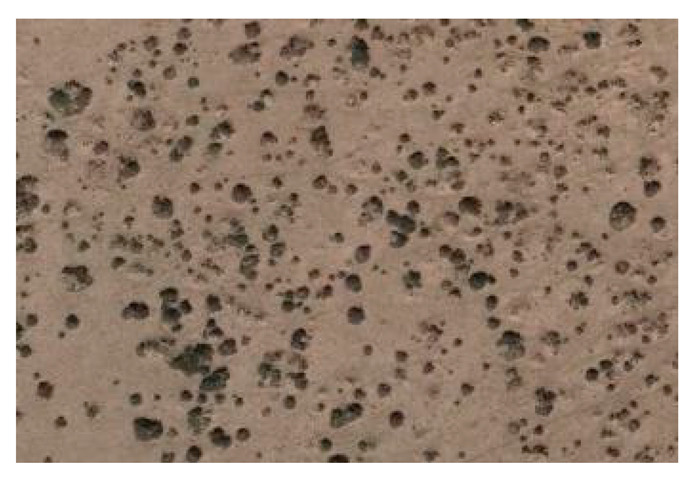	2–5	Branches are relatively slender, becoming tall and thin due to density	Some main stems are significantly inclined, and some are lodging	Mainly in the stable stage, with some in the activation stage

Note: The UAV aerial images illustrate the spatial distribution characteristics of the shrubs at the plot scale, while typical shrub photos are close-up images of individual shrubs taken from the ground. The evolutionary stages of shrubs were classified according to the criteria proposed by Du et al. [9]. Based on the morphological parameters of nabkhas that were obtained from the field investigations, combined with their morphological characteristics, the stages are qualitatively divided into development, stable, and activation stages.

**Table 2:** The classification criteria for soil particle size parameters.

Sorting Coefficient (σ_G_)	Skewness (SK_G_)	Kurtosis (K_G_)
Very well sorted (σ_G_ ≤ 0.35)	Very negatively skewed (−1.0 ≤ SK_G_ < −0.3)	Very platykurtic (K_G_ ≤ 0.67)
Well sorted (0.35 < σ_G_ ≤ 0.50)	Negatively skewed (−0.3 ≤ SK_G_ < −0.1)	Platykurtic (0.67 < K_G_ ≤ 0.90)
Moderately well sorted (0.50 < σ_G_ ≤ 0.71)	Near symmetrical (−0.1 ≤ SK_G_ < 0.1)	Mesokurtic (0.90 < K_G_ ≤ 1.11)
Moderately sorted (0.71 < σ_G_ ≤ 1.00)	Positively skewed (0.1 ≤ SK_G_ < 0.3)	Leptokurtic (1.11 < K_G_ ≤ 1.56)
Poorly sorted (1.00 < σ_G_ ≤ 2.00)	Very positively skewed (0.3 ≤ SK_G_ ≤ 1.0)	Very leptokurtic (1.56 < K_G_ ≤ 3.00)
Very poorly sorted (2.00 < σ_G_ ≤ 4.00)		Extremely leptokurtic (K_G_ > 3.00)
Extremely poorly sorted (σ_G_ > 4.00)		

**Table 3:** The morphological parameters of nabkhas under different shrub-cover plots.

Type	L_g_/cm	W_g_/cm	H_g_/cm	L_d_/cm	W_d_/cm	H_d_/cm	L_y_/cm	L_b_/cm
Control plots (CK)	64.80 ± 2.40 B	62.20 ± 3.20 B	22.27 ± 0.60 A	52.10 ± 4.50 A	47.40 ± 2.10 A	13.30 ± 0.40 A	36.00 ± 1.90 AB	26.20 ± 3.90 A
Low-cover plot (LCP)	67.40 ± 3.70 B	67.10 ± 0.90 AB	22.83 ± 0.30 A	58.00 ± 3.10 A	50.50 ± 1.10 A	13.00 ± 1.90 A	39.20 ± 1.50 A	29.90 ± 4.30 A
Medium-cover plot (MCP)	77.00 ± 3.30 A	68.90 ± 0.80 A	23.46 ± 0.50 A	58.20 ± 4.10 A	51.20 ± 2.90 A	13.30 ± 0.30 A	42.20 ± 2.50 A	30.10 ± 1.60 A
High-cover plot (HCP)	63.60 ± 1.80 B	63.50 ± 0.60 AB	22.36 ± 0.20 A	57.20 ± 2.20 A	48.40 ± 5.90 A	11.40 ± 0.50 A	30.50 ± 3.40 B	28.80 ± 2.50 A

Note: L_g_ denotes the shrub long axis, W_g_ denotes the shrub short axis, H_g_ denotes the shrub height, L_d_ denotes the nabkha long axis, W_d_ denotes the nabkha short axis, H_d_ denotes the nabkha height, L_y_ denotes the windward slope length, and L_b_ denotes the leeward slope length. The different uppercase letters indicate the significant differences among the plots with different shrub-cover levels (*p* < 0.05). All values were rounded to two decimal places; the same applies hereafter.

**Table 4:** The correlations between understory shrub cover and dune morphological parameters across plots with different coverage levels.

Type	Item	L_g_	W_g_	H_g_	L_d_	W_d_	H_d_	HC	S_d_	V_d_
Control plots (CK)	L_d_	0.82 **	0.64 **	0.44 **						
	W_d_	0.65 **	0.67 **	0.42 **	0.54 **					
	H_d_	0.76 **	0.62 **	0.29	0.74 **	0.46 **				
	HC	0.84 **	0.75 **	0.49 **	0.88 **	0.88 **	0.69 **			
	S_d_	0.76 **	0.69 **	0.46 **	0.78 **	0.92 **	0.62 **	0.97 **		
	V_d_	0.73 **	0.63 **	0.33 *	0.74 **	0.81 **	0.74 **	0.88 **	0.94 **	
	S_g_	0.91 **	0.97 **	0.48 **	0.64 **	0.64 **	0.65 **	0.73 **	0.68 **	0.66 **
Low-cover plot (LCP)	L_d_	0.07	0.19	0.13						
	W_d_	0.13	0.18	0.04	0.87 **					
	H_d_	0.08	0.13	−0.01	0.79 **	0.81 **				
	HC	0.10	0.19	0.09	0.97 **	0.96 **	0.83 **			
	S_d_	0.02	0.09	0.005	0.91 **	0.94 **	0.75 **	0.96 **		
	V_d_	−0.02	0.03	−0.06	0.85 **	0.91 **	0.74 **	0.91 **	0.94 **	
	S_g_	0.95 **	0.94 **	0.50 **	0.16	0.21	0.18	0.19	0.68 **	0.66 **
Medium-cover plot (MCP)	L_d_	0.24 **	0.22 *	0.10						
	W_d_	0.30 **	0.29 **	0.13	0.92 **					
	H_d_	0.08	0.09	0.07	0.33 **	0.29 **				
	HC	0.28 **	0.26 **	0.12	0.98 **	0.98 **	0.32 **			
	S_d_	0.22 *	0.19 *	0.06	0.92 **	0.90 **	0.28 **	0.93 **		
	V_d_	0.17	0.12	0.02	0.84 **	0.78 **	0.36 **	0.83 **	0.96 **	
	S_g_	0.94 **	0.92 **	0.53 **	0.15	0.23 *	0.04	0.19 *	0.15	0.11
High-cover plot (HCP)	L_d_	0.39 **	0.28 **	0.12						
	W_d_	0.29 **	0.28 **	0.06	0.85 **					
	H_d_	0.43 **	0.32 **	0.10	0.82 **	0.74 **				
	HC	0.35 **	0.29 **	0.10	0.96 **	0.96 **	0.81 **			
	S_d_	0.29 **	0.22 *	0.01	0.94 **	0.91 **	0.79 **	0.96 **		
	V_d_	0.30 **	0.20 *	−0.02	0.90 **	0.82 **	0.82 **	0.89 **	0.97 **	
	S_g_	0.96 **	0.93 **	0.51 **	0.33 **	0.27 **	0.39 **	0.31 **	0.27 **	0.28 **

Note: ** indicates significance at *p* < 0.01, * indicates significance at *p* < 0.05.

**Table 5:** The number and coverage proportion of nabkha dunes under different coverage plots.

Type	Number of Shrub Nabkhas	Vertical Projection Area Ratio (%)
Control plots (CK)	48	18.30%
Low-cover plot (LCP)	129	38.80%
Medium-cover plot (MCP)	209	57.20%
High-cover plot (HCP)	376	78.50%

## Data Availability

The data that support the findings of this study are available from the first and corresponding author upon reasonable request.

## References

[1] Tengberg A. (1995). Nebkha dunes as indicators of wind erosion and land degradation in the Sahel zone of Burkina Faso. J. Arid Environ..

[2] Tengberg A., Chen D.L. (1998). A comparative analysis of nebkhas in central Tunisia and northern Burkina Faso. Geomorphology.

[3] Nickling W.G., Wolfe S.A. (1994). The morphology and origin of nabkhas, region of Mopti, Mali, West Africa. J. Arid Environ..

[4] Hesp P., McLachlan A. (2000). Morphology, dynamics, ecology and fauna of *Arctotheca populifolia* and Gazania rigens nabkha dunes. J. Arid Environ..

[5] Yue X.L., Ha S., Zhuang Y.M., Zhuang J. (2005). Studies on sandy grassland nebkhas: A review. J. Desert Res..

[6] Yang F., Wang X.Q., He Q., Zhang X.Q., Han Z.Y., Huo W. (2014). Morphological features and spatial distribution pattern of *Tamarix ramosissima* nebkhas in an Oasis-desert ecotone. Arid Zone Res..

[7] Shabani S., Khaleel R. (2025). Evaluating the efficacy of fractal models for soil water retention curve estimation from particle-size distribution for Hanford sediments. Vadose Zone J..

[8] He Q.Q. (2024). Spatial Distribution Pattern, Morphological Characteristics and Depositional Pattern of *Tamarix ramosissima* Nebkhas in the Qira Oasis-Desert Ecotone. Master’s Thesis.

[9] Du J.H., Yan P., Ge Y.H. (2007). Distribution patterns and characteristics of *Nitraria tangutorun* nebkha at its different evolvement stages in the Minqin County of Gansu Province. Chin. J. Ecol..

[10] Han M., Gao Y., He M.H., Yan R., Bai F., Yang W.W., Li X.L., Yuan X.M., Yang J. (2024). Morphology and sediment characterization of a scrub sandpile in *Caragana tibetica*. J. Desert Res..

[11] Konert M., Vandenberghe J. (1997). Comparison of laser grain size analysis with pipette and sieve analysis: A solution for the underestimation of the clay fraction. Sedimentology.

[12] Yang W.Y., Gao Y., Han M., Yan R., Bai F. (2025). Particle size characteristics and soil physical and chemical properties of scrub sand pile soils in *Caragana tibetica*. North. Hortic..

[13] Ding G.D. (2010). Aeolian Physics.

[14] Folk R.L., Ward W.C. (1957). Brazos river bar: A study in the significance of grain size parameters. J. Sediment. Petrol..

[15] Chen X.C. (2016). The Migration Process of Aeolian Sand in Ulanbuh Desert Along the Dank of the Yellow River, Deng Kou. Master’s Thesis.

[16] Ding Y.L., Gao Y., Meng Z.J., Naren G., Huang X., Sun X.R., Wu H., Dang X.H., Wang M. (2016). Particle size characteristics of wind erosion surface soil in the desert steppe. Soils.

[17] Blott S.J., Pye K. (2001). GRADISTAT: A grain size distribution and statistics package for the analysis of unconsolidated sediments. Earth Surf. Process. Landf..

[18] Stoyan D., Stoyan H. (1994). Fractals, Random Shapes and Point Fields: Methods of Geometrical Statistics.

[19] Liu J., Xu X.Y., Zhang R.J., Ding A.Q., Fu G.Q., Zhao P. (2017). Spatial pattern of holes of *Rhombomys opimus* in A *Haloxylon ammodendron* plantation site. J. Desert Res..

[20] Gao Y., Dang X.H., Yu Y., Wang J., Wang S., Yuan W.J., Zhang X.W. (2015). Nabkha morphological characteristics and sand fixing capacity of *Artemisia sphaerocphalain* in the southeastern edge of the Ulan Buh Desert. J. Desert Res..

[21] Zhang P., Ha S., Yue X.L., Zhuang Y.M. (2008). Nitraria nebkhas: Morphological and sedimentary. Arid Land Geogr..

[22] Luo W.C., Zhao W.Z., Ren H., Liu B. (2021). Nebkha morphological characteristics and soil nutrition content in three regions with different climates in North China. J. Desert Res..

[23] Cabrera-Vega L.L., Cruz-Avero N., Hernández-Calvento L., Hernández-Cordero A.I., Fernández-Cabrera E. (2013). Morphological changes in dunes as an indicator of anthropogenic interferences in arid dune fields. J. Coast. Res..

[24] Zhang H.N., Lu X.H., Huang G.M., Li Y., Wang R.F., Luo Y.F., Liu J.L., Yang H. (2019). Morphological characteristics and spatial distribution patterns of *Vitextrifolia nebkhas* in the Poyang Lake sand land. Acta Ecol. Sin..

[25] Zhang P.J., Yang J., Song B.Y., Zhao L.Q., Qing H. (2009). Spatial heterogeneity of soil resources of *Caragana tibetica* communities. Chin. J. Plant Ecol..

[26] Zhang M. (2019). Morphological Features of *Caragana Tibetica* Nebkhas in Mu Us Desert. J. Chang. Norm. Univ..

[27] Qian Y.B., Zhang X.M., Li X.M. (1995). A study grain-size features of sand material of the oasis in the southern margin of the Taklimakan Desert. J. Desert Res..

[28] Zhang Z., Yu J., Wu L.J., Zhang Y.L., Li R. (2021). Sediment grain size characteristics and sedimentary environment significance in Hengshui area, Hebei Plain since 3.34 Ma. *Resour*. Environ. Arid Areas.

[29] Shi W.K., Dong Z.B., Liang A.M., Chen G.X., Liu R., Ma F. (2021). Grain-size characteristics and environmental significance of long ridge shaped Yardangs in the southeast Suoyang town, China. Resour. Environ. Arid Areas.

[30] Wu S.L., Li Z.Z., Jiao L., Hui J. (2008). Study on granulometric characteristics of sand material on Tamarix nabkha in the Hotan River Basin, Xinjiang. Arid Zone Res..

[31] Zhang J.R., Wang J.M., Zhu Y.C., Li B., Wang P. (2017). Research progress on the application of fractal theory in soil science. Chin. J. Soil Sci..

[32] Ma J.P., Wang F., Guo S.J., Ji Y.F., Zhang Y.H., Zhang Y.N., Zhang W.X., Song D.C. (2022). Fractal characteristics of soil particle size during *Nitraria tangutorum* shrub succession in Minqin. J. Northwest For. Univ..

[33] He X.X., Vmvt H., Dong Z.W., Asadilly Y., Alishir K. (2023). Spatial distribution pattern and intraspecific competition of *Populus euphratica* riparian forests under different water gradients. Acta Ecol. Sin..

[34] Li Q.Y., He Z.B., Zhao W.Z., Li Q.S. (2004). Spatial pattern of *Nitraria sphaerocarpa* populations and dynamic in different habitat. J. Desert Res..

[35] Yan H., Sun F.F., Ma S.M., Wang C.C., Zhang D., Zhang Y.L. (2021). Population structure and spatial distribution pattern of *Haloxylon ammodendron* and *H. persicum*. Southwest China J. Agric. Sci..

[36] Jia X.H., Li X.R. (2008). Spatial Pattern of Sand-Mound of *Nitraria* in Different Habitat at the Southeastern Fringe of the Tengger Desert. Environ. Sci..

[37] Su A.L., Xu G.P., Duan J.C., Wang S.P., Zhang Z.H. (2010). Community structure and point pattern analysis on main plant populations of *Potentilla fruticosa* shrub meadow in Qilian Mountain. Acta Bot. Boreali-Occident. Sin..

